# Rectum and Bladder Toxicity in Postoperative Prostate Bed Irradiation: Dose–Volume Parameters Analysis

**DOI:** 10.3390/cancers15225334

**Published:** 2023-11-09

**Authors:** Maja Hasterok, Monika Szołtysik, Zuzanna Nowicka, Bartłomiej Goc, Donata Gräupner, Wojciech Majewski, Konrad Rasławski, Paweł Rajwa, Iwona Jabłońska, Łukasz Magrowski, Mikołaj Przydacz, Wojciech Krajewski, Oliwia Masri, Marcin Miszczyk

**Affiliations:** 1 IIIrd Radiotherapy and Chemotherapy Department, Maria Skłodowska-Curie National Research Institute of Oncology, Wybrzeże Armii Krajowej 15, 44-102 Gliwice, Poland; maja.hasterok@gliwice.nio.gov.pl (M.H.); monika.szoltysik@gliwice.nio.gov.pl (M.S.); donata.graupner@gliwice.nio.gov.pl (D.G.); iwona.jablonska@gliwice.nio.gov.pl (I.J.); lukasz.magrowski@gliwice.nio.gov.pl (Ł.M.); oliwia.masri@gliwice.nio.gov.pl (O.M.); 2 Department of Biostatistics and Translational Medicine, Medical University of Lodz, Mazowiecka 15, 92-215 Lodz, Poland; zuzanna.nowicka@umed.lodz.pl; 3 Radiotherapy Department, Maria Skłodowska-Curie National Research Institute of Oncology, Wybrzeże Armii Krajowej 15, 44-102 Gliwice, Poland; bartlomiej.goc@gliwice.nio.gov.pl (B.G.); wojciech.majewski@gliwice.nio.gov.pl (W.M.); konrad.raslawski@gliwice.nio.gov.pl (K.R.); 4 Department of Urology, Medical University of Vienna, Währinger Gürtel 18-20, 1090 Vienna, Austria; pawelgrajwa@gmail.com; 5 Department of Urology, Medical University of Silesia, 3-go Maja 13-15, 41-800 Zabrze, Poland; 6 Department of Urology, Jagiellonian University Medical College, Macieja Jakubowskiego 2, 30-688 Krakow, Poland; mikolaj.przydacz@yahoo.com; 7 Department of Minimally Invasive and Robotic Urology, Wrocław Medical University, 50-556 Wrocław, Poland; wojciech.krajewski@umw.edu.pl

**Keywords:** salvage radiotherapy, adjuvant radiotherapy, postoperative irradiation, adverse events, prostate cancer, QUANTEC, dose–volume histogram, dose constraints

## Abstract

**Simple Summary:**

Postoperative radiotherapy can be associated with both early and late toxicity. In this retrospective analysis, we aimed to assess the prevalence of adverse events and their association with several dose–volume parameters. The results of our study showed that both rectum wall and rectum parameters within the range of 30–65 Gy presented significant association with acute ≥G2 gastrointestinal toxicity. On the other hand, the occurrence of late ≥G2 gastrointestinal toxicity was significantly associated only with rectum wall parameters, including V35–V54, V66–V69, and V71. Finally, the risk of experiencing late ≥G2 genitourinary adverse events was significantly associated with all bladder wall parameters between 30–54 Gy and all bladder parameters between 30–53 Gy. However, there was a clinically relevant risk of adverse events even in patients who adhered to commonly used dose constraints. This supports the conclusions of recent evidence showing that an early-salvage approach is preferable due to its non-inferior oncological outcomes.

**Abstract:**

Although prostate cancer treatment is increasingly effective, its toxicities pose a major concern. The aim of our study was to assess the rate of adverse events (AEs) and the prognostic value of dose–volume histogram (DVH) parameters for the occurrence of treatment toxicity in patients treated with post-prostatectomy prostate bed radiotherapy (RT). The AEs were scored according to the CTCAE v.5.0. The rectum and bladder were contoured according to the RTOG Guidelines. The DVH parameters were assessed using data exported from the ECLIPSE treatment-planning system. Genitourinary (GU) and gastrointestinal (GI) toxicity were analysed using consecutive dose thresholds for the percentage of an organ at risk (OAR) receiving a given dose and the QUANTEC dose constraints. A total of 213 patients were included in the final analysis. Acute grade 2 or higher (≥G2) GU AEs occurred in 18.7% and late in 21.3% of patients. Acute ≥G2 GI toxicity occurred in 11.7% and late ≥G2 in 11.2% of the patients. Five patients experienced grade 4 AEs. The most common adverse effects were diarrhoea, proctitis, cystitis, and dysuria. The most significant predictors of acute ≥G2 GI toxicity were rectum V47 and V46 (*p* < 0.001 and *p* < 0.001) and rectum wall V46 (*p* = 0.001), whereas the most significant predictors of late ≥G2 GI AEs were rectum wall V47 and V48 (*p* = 0.019 and *p* = 0.021). None of the bladder or bladder wall parameters was significantly associated with the risk of acute toxicity. The minimum doses to bladder wall (*p* = 0.004) and bladder (*p* = 0.005) were the most significant predictors of late ≥G2 GU toxicity. Postoperative radiotherapy is associated with a clinically relevant risk of AEs, which is associated with DVH parameters, and remains even in patients who fulfil commonly accepted dose constraints. Considering the lack of survival benefit of postoperative adjuvant RT, our results support delaying treatment through an early salvage approach to avoid or defer toxicity.

## 1. Introduction

Postoperative prostate bed irradiation can be applied either as an adjuvant treatment (aRT) due to adverse features, or as a salvage treatment (sRT) in cases of biochemical or local recurrence. Currently, aRT is rather uncommon, as it has been largely replaced by observation combined with early sRT in the majority of clinical scenarios [1,2,3]. However, it can still be offered to high-risk prostate cancer (PCa) patients who present in the International Society of Urological Pathology (ISUP) grade group 4–5 and pT3, or as a combination of aRT with adjuvant androgen deprivation therapy (ADT) in patients with pN1 disease after an extended lymph node dissection [4]. Both aRT and sRT are associated with a risk of early and late treatment toxicity, which may significantly deteriorate patients’ quality of life (QOL) [3].

The commonly found adverse events (AEs) fall into the gastrointestinal (GI) and genitourinary (GU) domains and include diarrhoea, rectal haemorrhage, proctitis, cystitis, urinary frequency, urgency, and incontinence, dysuria, or haematuria [3]. Their incidence is associated with various dose–volume parameters (DVPs) [5,6,7,8], which reflect the radiotherapy (RT) dose delivered to the organs at risk (OARs) contoured using RT planning computed tomography (CT). However, the DVPs show only the in silico treatment planning system (TPS) calculations; several factors can impact the reproducibility of DVP estimations in practice, including the interobserver variability in contouring and the geographical uncertainties associated with daily positioning [5,8].

In this study, we analysed the incidence of AEs in patients undergoing post-prostatectomy irradiation and the association of various DVPs with the occurrence of treatment toxicity based on real-world data.

## 2. Material and Methods

This retrospective analysis was based on data from patients treated with sRT or aRT, as described in an earlier publication [9]. The AEs were quantified retrospectively based on the patient’s medical history using the Common Terminology Criteria for Adverse Events (CTCAE) v5.0 grading system [10]. Each AE received an additional label, which classified the AEs as ‘genitourinary’, ‘gastrointestinal’, or ‘general’ (other). The OAR contours, including the bladder and rectum, were contoured anew according to the RTOG contouring guidelines [5]. Three-millimetre-wide rectum and bladder wall structures were created using the VARIAN ECLIPSE^TM^ TPS ‘extract wall’ function. Acute toxicity was defined as occurring within 90 days of the irradiation, and late toxicity as occurring thereafter. The dose–volume constraints (DVCs) proposed by Quantitative Analysis of Normal Tissue Effects in the Clinic (QUANTEC) [11,12] were used for the purpose of DVPs verification. Cumulative DVH parameters were used, representing the percentage or absolute volume receiving greater than or equal to the value in the corresponding dose bin. The parameter Vx used in our analysis represents the volume (%) of OAR receiving a dose of at least x Gy.

### Statistical Analysis

To find the dose levels that best predict toxicity, logistic regression was used to fit either GI or GU ≥ G2 toxicity as a response for each cut-off dose between 30 Gy and 77 Gy. This procedure was systematically repeated for acute and late toxicity outcomes. Models were fitted using ‘glm’ function from R package ‘stats’. The Kaplan–Meier curves, as implemented in Python 3 package ‘lifelines’, were used to plot cumulative probabilities of experiencing ≥G2 toxicity after postoperative prostate bed irradiation. *p* values < 0.05 were considered significant and all tests were 2-sided.

## 3. Results

The final analysis included 213 patients with a median age of 63.6 (interquartile range [IQR] 59.8–68.4) years who underwent RP between 1993 and 2013 and were later irradiated to the prostatic bed with either aRT (101; 47.4%) or sRT (112; 52.6%) between 2009 and 2014. Out of the initial database of 236 patients, 5 cases were excluded from the analysis due to missing TPS data, 17 cases due to missing toxicity data, and one patient due to a different fraction dose used in the treatment. The median RT dose was 70 Gy (range 62–76 Gy) in 2 Gy fractions, five times a week, using image-guided radiation therapy (IGRT) in all cases. The median FU was 61.4 months (IQR 38.1–78.4). Elective pelvic lymph node irradiation was performed in 58 (27.2%) patients using a standard dose of 44 Gy in 22 fractions. The majority of patients had pT2c stage 79 (37.1%), and 83 (39.0%) were administered ADT before RT. The detailed study group description is presented in Table 1.

### 3.1. Overall Treatment Toxicity

**Figure 1:**
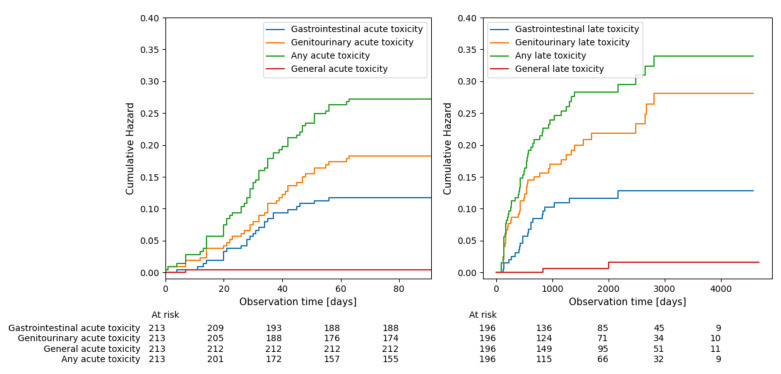
Cumulative hazard of experiencing >G2 toxicity estimated using Kaplan-Meier method..

We have recorded a total of 437 grade 1 (G1), 164 grade 2 (G2), 40 grade 3 (G3), and 5 grade 4 (G4) AEs, which are listed in Supplementary File, page 1. Acute ≥G2 GU AEs were found in 40 (18.7%), and acute ≥G2 GI AEs in 25 (11.7%) of 213 patients. One patient (0.5%) experienced other acute ≥G2 AE. Out of the 196 patients included in the late AEs analysis, 42 (21.3%) experienced ≥G2 GU AEs, and 22 (11.2%) ≥G2 GI AEs. Two (1.0%) patients experienced other ≥G2 late AEs. The cumulative hazard of ≥G2 AEs over time estimated using Kaplan-Meier method is presented in Figure 1. The actual incidence of radiotherapy-related toxicities is presented in Table 2.

The five G4 AEs included urinary tract obstruction, urinary fistula, sepsis, haematuria, and rectal haemorrhage, each of which was considered a Serious Adverse Effect (SAE) [13]. There was a total of 42 SAEs, most commonly associated with urinary tract obstructions, requiring intervention associated with hospitalisation (Supplementary File, page 2). The occurrence of acute ≥G2 GI toxicity did not predict late ≥G2 GI toxicity in the univariable analysis (odds ratio [OR] 0.91; 95% confidence interval [95%CI] 0.25–3.35; *p* = 0.895). The occurrence of acute ≥G2 GU toxicity did not predict late ≥G2 GU toxicity in the univariable analysis (OR 0.52; 95%CI 0.23–1.17; *p* = 0.895).

There was no significant difference between ≥G2 toxicity occurrence in patients treated with aRT or sRT. The time interval between RP and RT was not significantly associated with the occurrence of ≥G2 AEs (Supplementary File, page 3). Neither the time interval between RP and RT nor the radiotherapy modality used during the treatment was significantly associated with the occurrence of ≥G2 AEs (Supplementary File, pages 3 and 4).

### 3.2. Association between ≥G2 Toxicity Occurrence and Respective DVPs

Our study revealed no significant association between bladder or bladder wall parameters and the risk of developing acute ≥G2 GU toxicity. On the other hand, all bladder wall DVPs in the range of 30–54 Gy, as well as all bladder DVPs between 30–53 Gy, were found to be significantly associated with the risk of developing late ≥G2 GU AEs. According to our analysis, the minimum dose to the bladder wall (*p* = 0.004) and the minimum dose to the bladder (*p* = 0.005) were the most significant predictors of late ≥G2 GU toxicity.

All rectum wall DVPs in the range of 30–65 Gy, as well as all rectum DVPs in the range of 30–65 Gy, presented a significant association with the risk of developing acute toxicity. The most significant predictors were V47 and V46 for the rectum (*p* = 0.001 and *p* = 0.001, respectively) and V46 for the rectum wall (*p* = 0.001). The rectum wall DVPs, including V35-V54, V66-V69, and V71, were significantly associated with the risk of late ≥G2 GI toxicity, with rectum wall V47 and V48 (*p* = 0.019 and *p* = 0.021) being the most significant predictors. However, no significant association with late toxicity was observed for the corresponding rectum DVPs.

### 3.3. Association between QUANTEC DVCs and Organ Toxicity

Patients in whom the rectal DVCs exceeded the recommended limits had higher rates of acute and late ≥G2 GI toxicity, as shown in Table 3. It was not possible to estimate the association between exceeding QUANTEC DVCs for the bladder and acute or late ≥G2 GU toxicity, as only few patients included in our analysis exceeded the recommended DVCs (six, five, and three patients for V65 > 50%, V70 > 35%, and V75 > 25%, respectively).

The relationship between respective DVCs and ≥G2 toxicity rates for GU AEs could not be estimated. Only a few patients eligible for our study exceeded the recommended DVCs (six, five, and three patients for V65 > 50%, V70 > 35%, and V75 > 25%, respectively). Simultaneously, none of them experienced any GU-related AEs. Because of this, all patients with late ≥G2 GU toxicity fell within the “below constraint” group.

There was an association between late ≥G2 AEs and dose to respective wall parameters. For the GI tract, exceeding the V50 < 50% limit for the rectal wall led to an approximately threefold increase in the toxicity rate, as shown in Table 4. The ≥G2 GU toxicity was almost two times higher in patients whose minimum bladder wall dose exceeded 3% of the prescribed dose (Table 4). No similar associations were found for acute ≥G2 complications.

### 3.4. Association between ≥G2 Toxicity Occurrence and Clinical Parameters

Neither acute nor late ≥G2 GU and GI toxicity were significantly associated with patients’ comorbidities, including cardiovascular diseases, diabetes, and haemorrhoids. Similarly, there was no association between the use of hormonal therapy (HT), positive surgical margins, or age and toxicity (Supplementary File, page 5).

## 4. Discussion

AEs are relatively common in PCa patients undergoing sRT or aRT and have a negative influence on patients’ comfort [3]. The data on the prognostic value of dosimetric parameters in localised or locally advanced PCa treated with RT after RP are limited. In this article, we sought to investigate the real-world rates of clinically relevant AEs and their association with DVPs. We found that AEs are relatively common, and while there is a limited association between DVPs and acute ≥G2 GU toxicity, the parameters like bladder and bladder wall are important for (arguably more relevant) late urinary toxicity. Concerning the GI tract, rectum DVPs were found to be significant predictors for both acute and late ≥G2 AEs. Furthermore, the toxicity incidence is significantly associated with compliance with commonly used DVCs, such as the QUANTEC OAR dose constraints.

In comparison to the treatment toxicity profiles presented in the TROG 08.03/ANZUP RAVES [1], RADICALS-RT [2] and GETUG-AFU 17 [3] trials, our findings are consistent with regard to the more frequent incidence of ≥G2 GU toxicity in comparison to ≥G2 GI AEs [14]. Several authors have suggested that rectal toxicity tends to stabilise 2–3 years after RT [15,16,17]. In contrast, we found that GU toxicity increases over time and could be a major issue in the long term. The influence of many clinical factors [18,19,20,21] and the intensity of the acute inflammatory phase [15,22,23,24] on late toxicity have already been reported. However, in the case of our analysis, neither the comorbidities nor the intensity of acute toxicity was associated with the occurrence of late AEs related to both domains.

As expected, our results confirm that DVH parameters can effectively predict RT-related toxicity [20,25,26,27]. We found a significant correlation between late ≥G2 GU toxicity and parameters such as the bladder wall and bladder. Relatively similar findings of association between late ≥G2 GU toxicity and 51 Gy dose to the bladder have been reported in a study by Catucci et al. for patients with PCa treated with VMAT [28] and in the QUANTEC report [12]. Similarly, a prospective study by Schaake et al. demonstrated a significant relationship between specific scores of AEs and DVH parameters, including the bladder wall and the bladder’s trigone [29]. In another similar study, the dose range V40–V60 for the bladder was significantly correlated with late haematuria in patients receiving RT after RP, salvage or adjuvant [30]. In our analysis, the minimum dose to the bladder and bladder wall were the most significant predictors of late ≥G2 GU toxicity. This particular parameter might be significantly associated with the use of elective pelvic nodal irradiation. Low minimum doses are also easier to achieve with conformal RT, but it should not be interpreted in favour of older techniques, as low minimum doses are achieved at the expense of significant increases in other significant DVP domains.

We found that the rectal wall can be an important DVH structure. Lower doses to the rectal wall were associated with lower numbers of acute and late AEs, similar to previous studies [31,32,33,34]. Many studies reported an increased risk of late rectal bleeding when the rectum received higher RT doses [35,36,37]. Gulliford et al. reported an association between late GI AEs, such as rectal bleeding, proctitis, loose stools, and rectal urgency, and increasing dose in the range of 30–40 Gy to the rectum in patients treated with radical RT. For rectal bleeding and proctitis, the OR increased progressively from 20 Gy to 70 Gy, with the most significant prognostic value at 40 Gy threshold [38]. Another recent study showed that the risk of late rectal haemorrhage was significantly associated with doses in the range of V40 to V60 to the rectum [30].

Our findings confirmed that compliance with QUANTEC DVCs for the rectum results in a significant reduction in both acute and late GI toxicity rates in patients undergoing RT [12]. However, while there is space for improvement in terms of rectal sparing, almost all patients included in the analysis complied with the recommended dose limits for the bladder [11]. Importantly, adherence to the suggested constraints only reduced, but did not eliminate, the risk of experiencing various RT-induced AEs. This suggests the necessity to develop more modern, stricter dose constraints to match the increasing precision of modern RT techniques. This is yet another valuable argument for omitting unnecessary aRT in clinical scenarios where the early sRT approach provides equal clinical outcomes.

It should be noted that most of the generally recommended dose constraints were derived from the studies where RT was used as a definitive treatment [26,39,40], and data for aRT/sRT are scarce [4,41,42]. Investigating AEs in the postoperative setting, Shirai et al. [30] suggested dose constraints for avoiding late haematuria and rectal haemorrhage. According to the authors, meeting the suggested limits for the rectum (V60 ≤ 13%, V50 ≤ 33%) and bladder (V50 ≤ 43%, V40 ≤ 50%) may help reduce the incidence of respective G1 AEs [30], although such G1 toxicity is usually of little clinical importance. Compliance with the QUANTEC dose constraints resulted in an approximate 10% toxicity rate for both acute and late ≥G2 GI AEs in our study [12]. These findings were mainly based on conformal techniques, while the majority of our patients were treated with IMRT of VMAT. Several attempts have been made to define a new DVC better suited for modern RT techniques [26,39,43]. Fonteyene et al. proposed two sets of dose constraints for the rectum defined as its wall, which could allow to reduce the late ≥G2 rectal toxicity rate to ≤10% and ≤5% [43] respectively. In another study, Pederson et al. found that, among four different sets of constraints for three consecutive parameters (V40, V65, and V70), compliance with the strictest led to 100% freedom from ≥G2 GI toxicity (FFG2) at four years [26]. Those findings were later confirmed by Chennupati et al. for a larger group of patients. Moreover, patients compliant with these dose constraints reported higher GI-related QOL [39]. While QUANTEC constraints were intended for the bladder and rectum, defined as solid organs [11,12], there is a possible benefit in using wall parameters for hollow organs, which has already been described in previous studies [40,44]. Based on our findings, we believe that including additional DVCs for the bladder and rectum wall could help to further reduce late toxicity rates.

The main limitations of our study include the retrospective collection of data and the inhomogeneity of applied RT modalities. The analysis is subject to bias associated with the retrospective assessment of AEs, and the lack of data collected using standard questionnaires limits the assessment of QOL. The evolving RT planning methods might result in ‘fixed’ dose constraints becoming less relevant, and the toxicity profile schemes might differ for hypofractionated schedules. We were also unable to take into account the daily variability of OAR volumes, which restricts the reproducibility of the DVH assessment and might be compensated for in the future through the wide accessibility of automated adaptive RT solutions. Prospective studies on the optimal choice of DVCs in the setting of modern RT techniques, such as the combination of VMAT and adaptive RT, are necessary.

## 5. Conclusions

Prostate bed irradiation is associated with a clinically relevant risk of AEs. The risk of ≥G2 AEs can be reduced through adherence to DVCs, especially in terms of late toxicity, and the additional assessment of bladder and rectum wall parameters could improve the toxicity prediction of DVHs. However, even in patients adherent to the commonly accepted DVCs, the risk of AEs remains clinically relevant.

## Figures and Tables

**Table 1:** Clinical characteristics of the patients who underwent post-prostatectomy adjuvant or salvage radiotherapy.

**Number of Cases (All/Adjuvant/Salvage)**	N = 213	N = 101 (47.4%)	N = 112 (52.6%)
* Age [years]	63.6 (59.8–68.4)	62.3 (58.8–66.3)	65.2 (60.5–70.2)
* Follow-up [months]	61.4 (38.1–78.4)	64.9 (38.1–86.6)	57.6 (38.5–74.4)
* Time from RP ^1^ to RT ^2^ [months]	6.2 (3.4–25.8)	3.7 (3.0–4.8)	22.6 (7.0–46.2)
**Total dose (prostate bed) [Gy]**	70. (70–74); Min 62, Max 76	70 (70–72); Min 62, Max 76	74 (70–76); Min 66, Max 76
* max PSA pre-RP [ng/mL]	9.24 (6.75–14.61)	8.87 (6.70–14.39)	10.33 (7.00–14.69)
* max PSA post-RP [ng/mL]	0.27 (0.04–1.14)	0.03 (0.01–0.10)	1.00 (0.40–2.38)
* pT stage **pT2a** **pT2b** **pT2c** **pT3a** **pT3b**	32 (15%) 12 (5.6%) 79 (37.1%) 39 (18.3%) 44 (20.7%)	6 (5.9%) 4 (4.0%) 39 (38.6%) 27 (26.7%) 24 (23.8%)	26 (23.2%) 8 (7.1%) 40 (35.7%) 12 (10.7%) 20 (17.9%)
**ADT ^3^** **(receiving)**	83 (39.0%)	35 (34.7%)	48 (42.9%)
**ISUP ^4^ grade group**			
**1**	83 (39.0%)	33 (32.7%)	50 (44.6%)
**2**	72 (33.8%)	41 (40.6%)	31 (27.7%)
**3**	31 (14.6%)	14 (13.9%)	17 (15.2%)
**4**	12 (5.6%)	5 (5.0%)	7 (6.3%)
**5**	11 (5.2%)	7 (6.9%)	4 (3.6%)
**Radiotherapy modality ^**			
**IMRT ^5^**	117 (54.9%)	58 (57.4%)	59 (52.7%)
**3DCRT ^6^**	38 (17.8%)	16 (15.8%)	22 (19.6%)
**VMAT ^7^**	35 (16.4%)	16 (15.8%)	19 (17.0%)
**Tomotherapy**	8 (3.8%)	3 (3.0%)	5 (4.5%)
**IMRT + VMAT**	6 (2.8%)	3 (3.0%)	3 (2.7%)
**3DCRT + IMRT**	5 (2.3%)	3 (3.0%)	2 (1.8%)
**3DCRT + VMAT**	4 (1.9%)	2 (2.0%)	2 (1.8%)
**Pelvic lymph node irradiation**	58 (27.2%)	33 (32.7%)	25 (22.3%)

* Data presented as: median (IQR); ^1^ RP—radical prostatectomy; ^2^ RT—radiotherapy; ^3^ ADT—androgen deprivation therapy; ^4^ International Society of Urological Pathology grade group; ^ Different modality used for prostate bed and elective pelvic irradiation; ^5^ IMRT—intensity modulated radiation therapy; ^6^ 3DCRT—3D conformal radiation therapy; ^7^ VMAT—volumetric modulated arc therapy.

**Table 2:** Total number of patients reporting ≥G2 toxicities.

	N (%)
grade ≥ 2 GI acute	25 (11.7%)
grade ≥ 2 GU acute	40 (18.7%)
grade ≥ 2 other acute	1 (0.5%)
grade ≥ 2 GI late	22 (11.2%)
grade ≥ 2 GU late	42 (21.3%)
grade ≥ 2 other late	2 (1.0%)

**Table 3:** Relationship between QUANTEC DVCs and acute or late ≥G2 toxicity rates for GI tract.

DVCs						
Rectum	V50 < 50%	V50 > 50%	V70 < 35%	V70 > 35%	V75 < 25%	V75 > 25%
**≥G2 acute toxicity rate**	10.3%	26.3%	11.3%	15.8%	11.1%	16.7%
**≥G2 late toxicity rate**	9.3%	21.1%	9.8%	15.8%	8.5%	25.0%

DVCs—dose–volume constraints. GI—gastrointestinal. Vx > x%—Vx represents the volume (%) of OAR receiving a dose of at least x Gy.

**Table 4:** Relationship between selected rectum and bladder wall dose parameters and respective late ≥G2 toxicity.

	DVCs	
**Rectum wall**	**V50 < 50%**	**V50 > 50%**
**≥** **G2 late toxicity rate**	9.1%	28.6%
**Bladder wall**	**Dmin < 3%**	**Dmin > 3%**
**≥** **G2 late toxicity rate**	14.7%	28.7%

DVCs—dose–volume constraints.

## Data Availability

Additional data are presented in the Supplementary File. Specific anonymized data can be shared by the authors upon reasonable request.

## Supplementary Materials

The following supporting information can be downloaded at: https://www.mdpi.com/article/10.3390/cancers15225334/s1, Supplementary File consists of the Supplementary Tables S1–S6. Table S1: Summary of all recorded treatment toxicities with respective grades; Table S2: Summary of RT—related ≥G2 Serious Adverse Effects; Table S3: ≥G2 toxicity occurrence in patients treated with aRT compared to sRT; Table S4: Association between ≥G2 toxicity occurrence and time from RP to RT. Table S5: Association between ≥G2 toxicity occurrence and radiotherapy modality used; Table S6: Clinical description of the study group.
