# Supplementary material for: Rectum and Bladder Toxicity in Postoperative Prostate Bed Irradiation: Dose–Volume Parameters Analysis
Source: Cancers (Basel). 2023 Nov 9;15(22):5334. doi: 10.3390/cancers15225334 (PMC10670737; doi:10.3390/cancers15225334)
Supplement: Supplementary file 1 [file cancers-15-05334-s001.zip › cancers-2706679-supplementary.pdf]

SUPPLEMENT

TABLE S1. SUMMARY OF ALL RECORDED TREATMENT TOXICITIES WITH RESPECTIVE GRADES

GRADE	NUMBER OF AES: N(%)	TYPE OF AES : N(%)			
**G1**	437 (67.6%)	Cystitis noninfective	**75 (17.2%)**	Nausea	**3 (0.7%)**
		Diarrhea	**73 (16.7%)**	Fatigue	**11 (2.5%)**
		Urinary urgency	**10 (2.3%)**	Testicular pain	**2 (0.5%)**
		Urinary frequency	**71 (16.2%)**	Penile pain	**1 (0.2%)**
		Urinary incontinence	**12 (2.7%)**	Urinary tract pain	**4 (0.9%)**
		Dysuria	**18 (4.1%)**	Dysaesthesia	**1 (0.2%)**
		Urinary tract obstruction	**25 (5.7%)**	Headache	**1 (0.2%)**
		Proctitis	**48 (11%)**	Back pain	**2 (0.5%)**
		Constipation	**31 (7.1%)**	Rectal pain	**1 (0.2%)**
		Abdominal pain	**15 (3.4%)**	Pelvic pain	**2 (0.5%)**
		Pain	**1 (0.2%)**	Insomnia	**1 (0.2%)**
		Haematuria	**2 (0.5%)**	Flatulence	**6 (1.4%)**
		Rectal haemorrhage	**21 (4.8%)**		
**G2**	164 (25.4%)	Rectal haemorrhage	**5 (3%)**	Haematuria	**2 (1.2%)**
		Urinary frequency	**3 (1.8%)**	Abdominal pain	**2 (1.2%)**
		Urinary tract infection	**38 (23.2%)**	Urethral infection	**2 (1.2%)**
		Urinary incontinence	**13 (7.9%)**	Constipation	**1 (0.6%)**
		Proctitis	**35 (21.3%)**	Flatulence	**1 (0.6%)**
		Cystitis noninfective	**26 (15.9%)**	Fecal incontinence	**1 (0.6%)**
		Diarrhea	**7 (4.3%)**	Urinary tract pain	**3 (1.8%)**
		Urinary tract obstruction	**23 (14%)**	Penile infection	**1 (0.6%)**
		Shingles	**1 (0.6%)**		
**G3**	40 (6.2%)	Urinary tract obstruction	**26 (65%)**	Proctitis	**1 (2.5%)**
				Urinary tract infection	**1 (2.5%)**
		Urinary incontinence	**2 (5%)**		
		Shingles	**1 (2.5%)**	Anaemia	**1 (2.5%)**
		Diarrhea	**4 (10%)**	Anorectal infection	**1 (2.5%)**
		Urinary fistula	**1(2.5%)**		
		Cystitis noninfective	**2 (5%)**		
**G4**	5 (0.8%)	Urinary tract obstruction<br>Urinary fistula<br>Sepsis<br>Haematuria<br>Rectal haemorrhage		**All 1 (20%)**	

TABLE S2. SUMMARY OF RT – RELATED ≥G2 SERIOUS ADVERSE EFFECTS

TYPE OF AES	N(%)
Urinary tract obstruction	29 (69%)
Urinary incontinence	1 (2.4%)
Urinary fistula	2 (4.8%)
Sepsis	1 (2.4%)
Cystitis noninfective	2 (4.8%)
Haematuria	1 (2.4%)
Proctitis	1 (2.4%)
Urinary tract infection	1 (2.4%)
Rectal haemorrhage	1 (2.4%)
Diarrhea	1 (2.4%)
Anaemia	1 (2.4%)
Anorectal infection	1 (2.4%)

TABLE S3. ≥G2 TOXICITY OCCURRENCE IN PATIENTS TREATED WITH ART COMPARED TO SRT

≥G2 TOXICITY IN ART VS SRT GROUPS	HR*	95% CI FOR HR	P VALUE
**acute GI**	1.72	(0.77 – 3.83)	0.18
**acute GU**	0.93	(0.49 – 1.74)	0.81
**late GI**	0.81	(0.35 – 1.90)	0.63
**late GU**	1.28	(0.70 – 2.35)	0.42

* **REFERENCE POINT-ART**

TABLE S4. ASSOCIATION BETWEEN ≥G2 TOXICITY OCCURRENCE AND TIME FROM RP TO RT

≥G2 TOXICITY AND TIME TO RT	HR*	95% CI FOR HR	P VALUE
**acute GI**	0.98	(0.96 – 1.01)	0.15
**acute GU**	1.01	(1.00 – 1.01)	0.20
**late GI**	1.00	(0.98 – 1.01)	0.59
**late GU**	0.99	(0.97 – 1.00)	0.16

* **REFERENCE POINT-RT**

TABLE S5. ASSOCIATION BETWEEN ≥G2 TOXICITY OCCURRENCE AND RADIOTHERAPY MODALITY USED

≥G2 TOXICITY IN DYNAMIC VS STATIC RT METHOD	HR	95% CI FOR HR	P VALUE
**acute GI**	0.69	(0.29 – 1.66)	0.41
**acute GU**	1.68	(0.70 – 4.01)	0.24
**late GI**	1.50	(0.51 – 4.44)	0.46
**late GU**	0.91	(0.46 – 1.82)	0.79

Dynamic: IMRT, VMAT, Tomotherapy
Static: 3DCRT, 3DCRT+VMAT, 3DCRT+IMRT

TABLE S6. CLINICAL DESCRIPTION OF THE STUDY GROUP

≥G2 TOXICITY	CARDIOVASCULAR DISEASES		DIABETES		HAEMORRHOIDS		HT BEFORE RT		R1 SURGICAL MARGIN	
	**OR (95% CI)**	**P VALUE**	**OR (95% CI)**	**P VALUE**	**OR (95% CI**	**P VALUE**	**OR (95% CI)**	**P VALUE**	**OR (95% CI)**	**P VALUE**
**acute GI**	0.88 (0.35-2.23)	0.978	1.53 (0.32-7.37)	0.639	4.81 (0.82-28.08)	0.115	1.15 (0.48-2.75)	0.746	1.1 (0.46-2.63)	0.998
**acute GU**	1.56 (0.73-3.32)	0.335	0.79 (0.17-3.69)	1	5.16 (1.0-26.76)	0.065	1.44 (0.71-2.90)	0.310	1.28 (0.6-2.72)	0.658
**late GI**	0.57 (0.22-1.48)	0.357	0.26 (0.02-4.61)	0.371	1.65 (0.18-14.9)	0.508	0.71 (0.28-1.83)	0.479	1.07 (0.42-2.73)	0.929
**late GU**	1.02 (0.5-2.09)	0.952	2.27 (0.71-7.23)	0.28	4.03 (0.78-20.82)	0.106	0.96 (0.48-1.95)	0.919	1.34 (0.65-2.77)	0.533

GI – gastrointestinal; GU – genitourinary; OR – odds ratio; CI – confidence interval
